# The transcriptional profiles and functional implications of long non-coding RNAs in the unfolded protein response

**DOI:** 10.1038/s41598-018-23289-3

**Published:** 2018-03-21

**Authors:** Hongyang Quan, Qianqian Fan, Chuang Li, Yan-ying Wang, Lin Wang

**Affiliations:** 1 Department of Physiology, Institute of Basic Medical Sciences, School of Basic Medicine Peking Union Medical College, Chinese Academy of Medical Sciences, 5 Dong Dan San Tiao, Beijing, 100005 China; 2 Present Address: Patent Examination Corporation, State Intellectual Property Office, 2028 Tianfu Avenue South, Chengdu, 610213 China; 3 Present Address: National Research Institute for Family Planning, 12 Da Hui Si, Beijing, 100080 China

## Abstract

The unfolded protein response (UPR) is activated, when the folding capacity is compromised in the endoplasmic reticulum (ER). To date, most studies focused on the coding genes and microRNAs in UPR. Other non-coding RNAs affected by UPR and their roles in UPR have not been systematically studied. Long noncoding RNAs (lncRNAs) are increasingly recognized as powerful epigenetic regulators. In this study, we transcriptomically profiled the lncRNAs and mRNAs from mouse embryonic fibroblasts under ER stress, and identified many differentially expressed lncRNAs and mRNAs. Genomic location and mRNA-lncRNA co-expression analyses predicted a number of lncRNAs, which potentially regulate the expression of UPR genes. In particular, FR229754, an exonic sense lncRNA, is significantly up-regulated in UPR. FR229754 overlaps with *Sel1l*, and their expressions correlated with each other. Sel1l is involved in the ER-associated protein degradation. Silencing of FR229754 did not much affect the expression of *Sel1l*, but markedly reduced the levels of *BiP/GRP78/Hspa5*, a major ER chaperon up-regulated in UPR. Probing with pathway-specific inhibitors showed that up-regulation of FR229754 and *Sel1* depended on the activation of PERK. Together, our study identified a number of candidate lncRNAs and paved the way for future characterization of their functions in UPR.

## Introduction

In eukaryotic cells, the endoplasmic reticulum (ER) is a major organelle that the secretory and membrane proteins enter the secretory pathway to fold, modify and mature. Environmental, physiological and pathophysiological conditions may alter the flux of proteins into the ER and exceed its folding capacity. The resulted accumulation of unfolded proteins activates multiple adaptive cellular processes, which are collectively known as ER stress response and include the unfolded protein response (UPR)^1^. UPR originally protects the cells to survive the stress insult, and could commit the cells to apoptosis, if the adaptive responses fail to restore the ER proteostasis.

In mammalian cells, UPR encompasses three branches, namely the inositol-requiring protein-1α (IRE1α), activating transcription factor-6α (ATF6α) and protein kinase RNA (PKR)-like ER kinase (PERK)^2,3^. In the unstressed cells, IRE1α, ATF6α and PERK are bound to BiP/GRP78/Hspa5, a major chaperon in the ER lumen. Upon ER stress, BiP binds to the unfolded proteins accumulated in the ER lumen and dissociates from the three UPR sensors. IRE1α, ATF6α and PERK are then activated by a series of events, including dimerization, phosphorylation, endonuclease splicing, ER-Golgi trafficking and proteolytic processing, and generate three respective downstream transcription factors, namely X-box binding protein 1(XBP1), activating transcription factor 4 (ATF4) and ATF6α. XBP1, ATF4 and ATF6α signal the nucleus to increase the ER chaperon expression to assist protein folding, the phospholipid synthesis to expand the ER, and the ER-associated protein degradation (ERAD) to remove the unfolded proteins through the ubiquitin proteasome system^2–4^.

UPR is regulated by multiple cellular mechanisms including microRNAs. Dozens of microRNAs are differentially expressed after the treatment with ER stress inducers, such as tunicamycin and thapsigargin. Several differentially expressed microRNAs also function as modulators of UPR. For example, miR-30c-2* is up-regulated in UPR by PERK, and in turn attenuates UPR by decreasing XBP1^5^. MiR-211, which is also induced after PERK activation, represses the CHOP transcription by targeting its 5′ untranslated region^6^. Some microRNAs can affect the expression of UPR-unrelated proteins. For example, miR-708, which is transcribed from an intron of a CHOP-regulated gene, *Odz4*, controls the expression of rhodopsin in retina and prevents it from entering the ER^7^.

In the past decade, long noncoding RNAs (lncRNAs) have emerged as another class of non-protein coding RNAs, which play important regulatory roles in multiple cellular processes^8^. lncRNAs can be classified as intronic, exonic, overlapping and intergenic lncRNAs, based on their locations in regard to the nearest protein-coding genes^9^. Exonic lncRNAs can be further classified as sense and antisense lncRNAs based on the direction of transcription. The long intergenic noncoding RNAs, also known as lincRNAs, are found to contain introns, exons and polyadenylated tails similarly as the mRNAs, and can be spliced to different transcripts^10,11^.

Mechanistically, lncRNAs regulate gene transcription either in *cis* or in *trans*. For example, Xist, which is transcribed from one X chromosome, activities the X chromosome silencing^12–14^. HOTAIR, which is transcribed from the HOXC cluster, interacts with the Polycomb repressive complex 2 to represses the transcription of the HOXD cluster in *trans*^15,16^. Some lncRNAs post-transcriptionally regulate genes expression by affecting the pre-mRNA splicing and mRNA translation. MALAT1, a nuclear lncRNA widely associated with cancer metastasis, affects the phosphorylation of serine/arginine-containing splicing factors^17^. lncRNAs can also function as competing endogenous RNAs (ceRNAs) to interact with microRNAs and antagonize the microRNA-mediated regulation. For example, HULC, a lncRNA highly up-regulated in liver cancers, can act as a ceRNA to sponge miR-372, and reduce the transcriptional repression of miR-372 targets genes^18^.

In addition to the microRNAs affected by or modulating UPR, several lncRNAs have also been linked with UPR^19^. The expression of calreticulin, an ER chaperone involved in glycoprotein folding, is regulated by miR-455 and ncRNA-RB1, a lncRNA that shares a bidirectional promoter with the RB1 gene. Silencing of ncRNA-RB1 reduced calreticulin levels^20^. Malat1 was reported to be up-regulated about two fold in UPR, and the up-regulation depended on PERK activation^21^. A megacluster of microRNAs and their host long non-coding RNA transcript (lnc-MGC) are increased in the glomeruli of mouse models of diabetic nephropathy^22^. Lnc-MGC appears to be regulated by CHOP, a transcription factor downstream of PERK. Lnc-MGC plays a role in glomerular extracellular matrix formation and hypertrophy in diabetic mice, possibly through interaction with the cluster microRNAs. Hypoxia significantly increased the expression of HypERlnc, a lncRNA in pericytes and perivascular mural cells^23^. HypERlnc has been suggested to regulate both the viability and permeability of pericytes and endothelial cells and UPR activation, which is believed to contribute to the development of many cardiopulmonary diseases.

To date, the global expression profile of lncRNAs under ER stress has not been reported. In this study, we used a comprehensive mRNA/lncRNA microarray to examine the transcriptome of MEFs under ER stress, and identified a large number of differentially expressed lncRNAs and mRNAs. We then performed genomic location and mRNA-lncRNA co-expression analyses to identify several candidate lncRNAs, which potentially regulate the expression of UPR genes. In particular, we carried out characterization of the functional involvement and expression of FR229754, an up-regulated lncRNA, in UPR.

## Results

### Time course of UPR activation in MEFs

**Figure 1:**
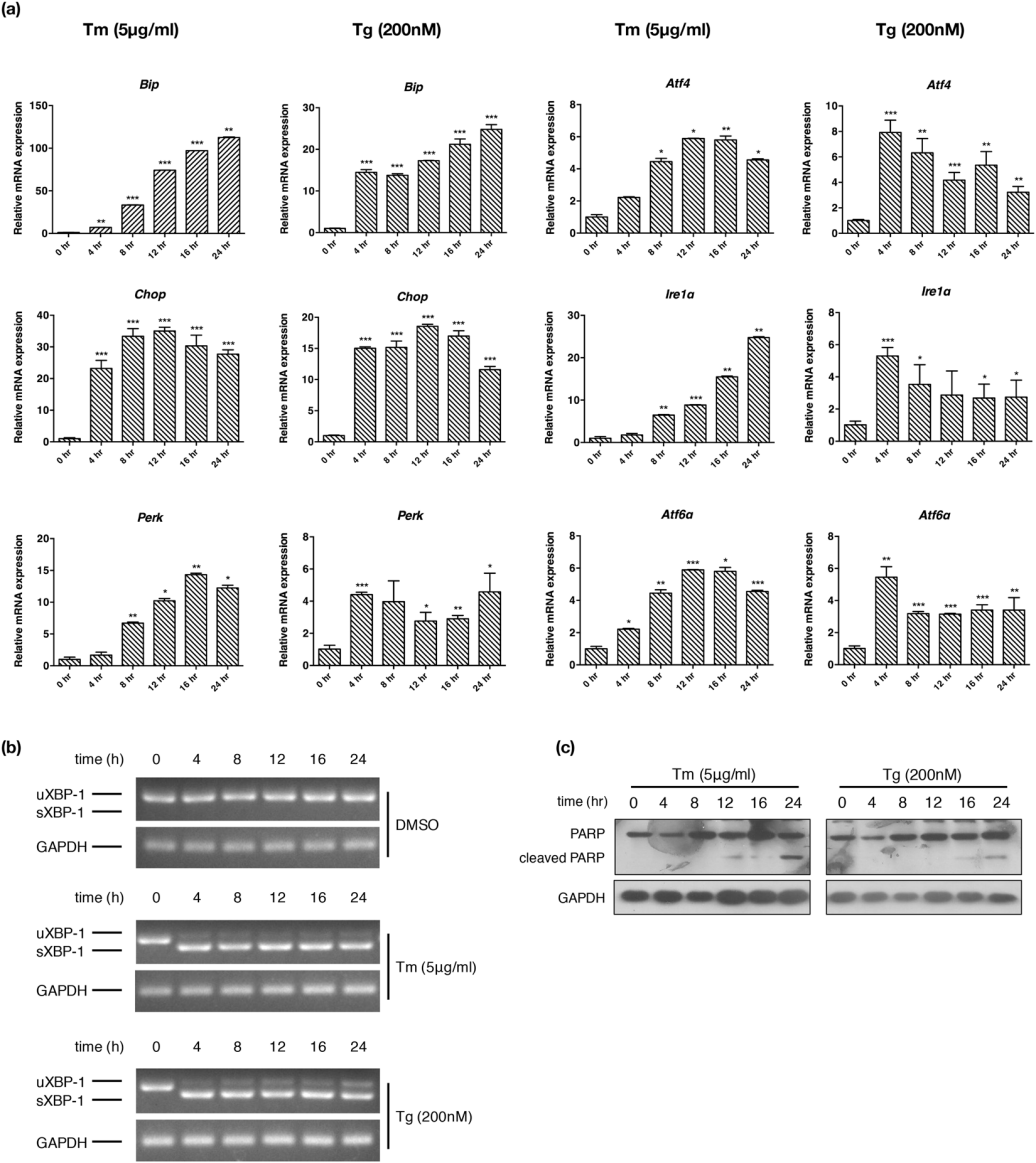
Time course of UPR activation in MEFs under ER stress. (**a**) MEFs were treated with 5 μg/ml tunicamycin (Tm), 200 nM thapsigargin (Tg) and DMSO as a vehicle control for the indicated time, and the expression of UPR (*BiP*, *Atf4*, *Chop, Ire1*α, *Perk*, and *Atf6α)* genes were analyzed by real-time PCR. All examined RNAs were normalized to *Gapdh*. (**b**) *Xbp-1* mRNA splicing was determined by reverse transcription-PCR. Unspliced (u) and spliced (s) *Xbp-1* mRNA products are indicated. *Gapdh* PCR products serve as a loading control. (**c**) Immunoblotting analysis of PARP and its cleaved fragment. Gapdh serves as a loading control. Data are shown as mean + SD. *P < 0.05, **P < 0.01 and ***P < 0.001. n = 3.

Before performing microarray analysis, we first determined the time course of the UPR activation in MEFs. The MEFs were treated with 5 μg/ml tunicamycin, 200 nM thapsigargin or vehicle control for up to 24 h, and the cells were harvested at 0, 4, 8, 12, 16, 24 h, respectively. Real-time PCR experiments showed that the expression of *BiP*, *IRE1α*, *PERK*, ATF6*α*, *Chop* and ATF4, another transcription factor downstream of PERK, steadily increased over 24 h (Fig. 1a). The expression of *IRE1β* and ATF6*β*, the isoforms of *IRE1α* and ATF6*α* that are not activated in UPR, remained largely unchanged (Supplementary Fig. 1). Analysis of *Xbp-1* splicing showed that *Xbp-*1 became fully spliced, which indicated the activation of the IRE1α branch, as early as 4 h (Fig. 1b). These results together demonstrated that the transcriptional up-regulation of many UPR target genes increased over 24 h in MEFs, and the three branches were likely activated in different chronological orders, a finding consistent with several previous reports^24–26^. Because prolonged UPR activation is known to trigger apoptosis, we also examined the cleavage of poly (ADP-ribose) polymerase (PARP) in MEFs treated with tunicamycin and thapsigargin. Much lower or undetectable PARP cleavage was seen in the MEFs treated with tunicamycin or thapsigargin for 4 to 16 h, but the cleavage was significantly increased in the MEFs with UPR induced for 24 h, indicating that apoptosis was minimal at 16 h under these conditions (Fig. 1c). Therefore we chose 16 h as an arbitrary time point to analyze the expression profile of mRNAs and lncRNAs from the tunicamycin-treated MEFs.

### Microarray analysis of lncRNA and mRNA profiles in MEFs

**Figure 2:**
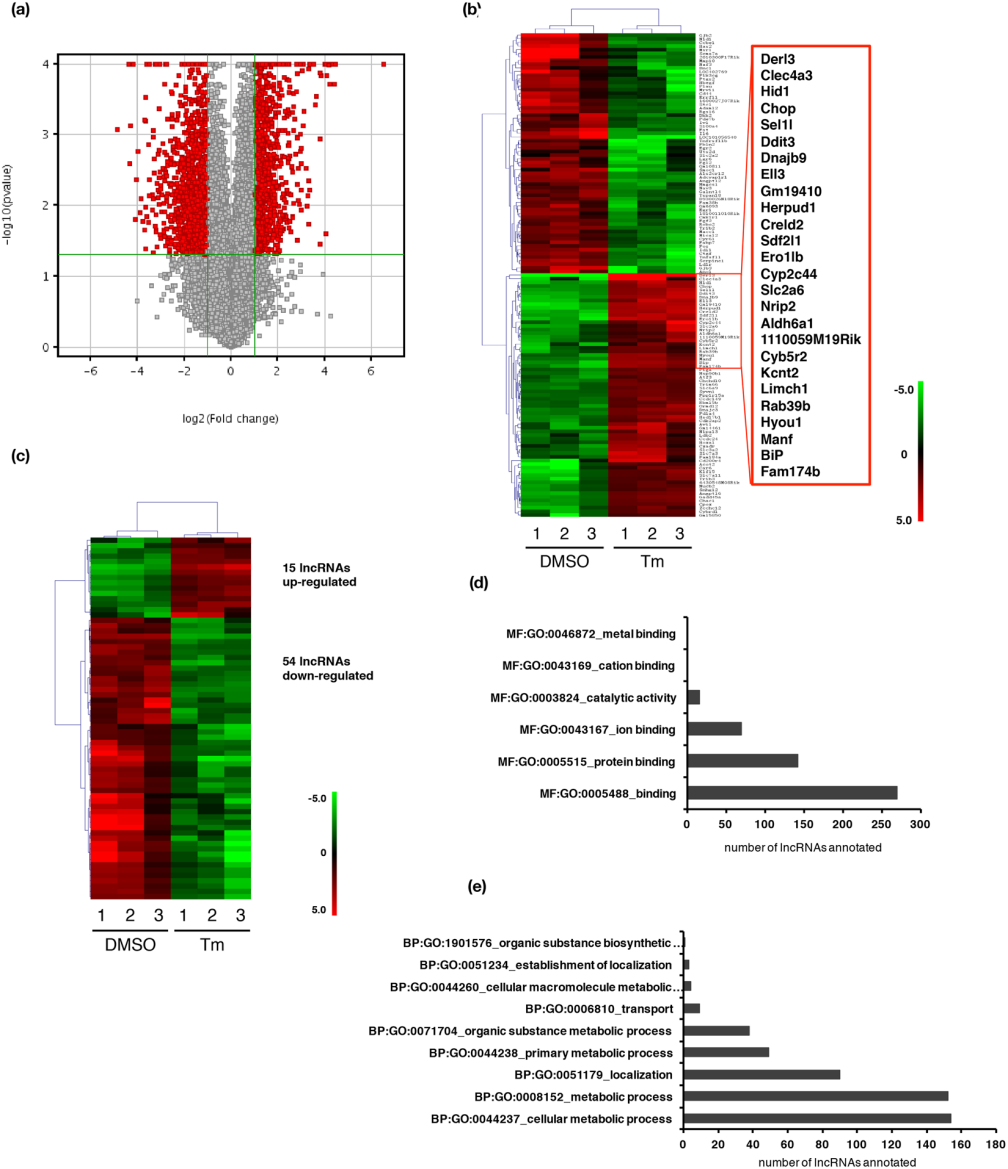
Microarray analysis of mRNAs and lncRNAs in MEFs under ER stress. (**a**) Volcano plot of the lncRNA expression levels between the control and tunicamycin (Tm)-treated MEFs. (**b,c**) Hierarchical clustering heat maps showing mRNAs and lncRNAs changed over 5 fold between DMSO (control) and Tm-treated MEFs. The zoom-in window in (**b**) shows the list of mRNAs up-regulated over tenfold. The 1,2,3 represent three individual samples of DMSO- and Tm-treated MEFs. (**d**) The possible molecular functions of the most significantly changed 500 lncRNAs predicted by lncRNA-mRNA co-expression analysis. (**e**) The possible biological processes that these lncRNAs are involved in.

Our microarray contains 51,302 probes, which were 60-nucleotide long and designed to hybridize with the entire mouse transcriptome of 24,239 mRNAs and 35,757 lncRNAs. The mouse lncRNAs were pooled from the several lncRNA databases: Ensembl, RefSeq, Ultra-conserved region encoding LncRNA (UCR), lncRNAdb, ncRNA and NONCODE. In our study, the expression of 881, 69 and 13 lncRNAs, and 976, 129 and 32 mRNAs was found to change over 2, 5 and 10 fold in the tunicamycin-treated MEFs, respectively (Fig. 2a). The mRNAs and lncRNAs changed over 5 fold are shown in heatmaps (Fig. 2b,c). *Chop*, *Herpud1* (*homocysteine inducible er protein with ubiquitin like domain 1*)*, Ero1lb* (*endoplasmic reticulum oxidoreductase 1β*), *BiP* and *Hrd1*/*Snyn1* (*HMG-CoA reductase degradation 1/synoviolin 1*) were among the most strongly up-regulated genes (DEGs) (Fig. 2b). 15 and 54 lncRNAs increased or decreased over 5 fold, respectively, and they were further categorized into 13 intergenic, 18 intronic, 32 exonic sense, 2 exonic antisense and 4 overlapping lncRNAs.

**Figure 3:**
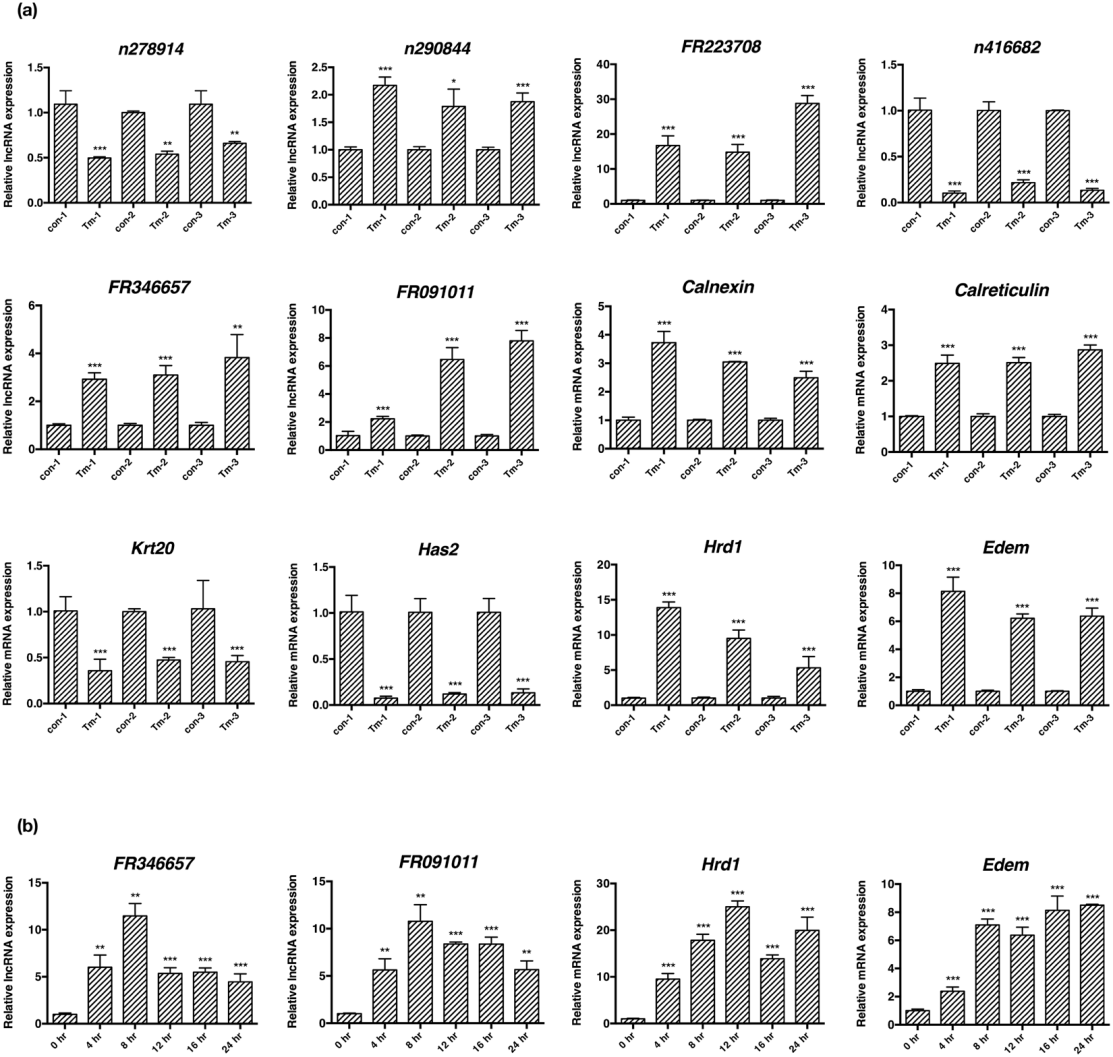
Validation of mRNA and lncRNA expression in MEFs under ER stress. (**a**) Real-time PCR analysis of the expression of six randomly selected lncRNAs and mRNAs from three independent sets of DMSO (control) and tunicamycin (Tm)-treated MEFs at 16 h. (**b**) The expression of FR346657, FR091011, *Hrd1* and *Edem1* over the course of UPR for up to 24 h as analyzed by real-time PCR analysis. All examined RNAs were normalized to *Gapdh*. Data are shown as mean + SD. *P < 0.05, **P < 0.01 and ***P < 0.001. n = 3.

The top 500 differentially expressed lncRNAs are predicted to be involved in several molecular functions, namely protein binding, ion binding and catalytic activity, and participate in a host of biological processes: metabolism, localization biosynthesis and transport (Fig. 2d,e). We then used real-time PCR to validate the expression of six randomly selected lncRNAs (n278914, n290844, FR223708, n416682, FR346657 and FR091011) and mRNAs (*Calnexin*, *Calreticulin*, *Krt20*, *Has2*, *Hrd1* and *Edem*), which were differentially expressed after UPR activation as identified by the microarray study. Comparable trend of change was seen by both techniques from the RNAs prepared from three independently cultured MEFs (Fig. 3a). Furthermore, we examined the time course of FR346657, FR091011, *Hrd1* and *Edem* expression in MEFs treated with 5 μg/ml tunicamycin for up to 24 h (Fig. 3b). Most of their expression reached planteu at 8 h, and subsequently decreased or maintained throughout 24 h.

### Genomic locations of the differentially expressed lncRNAs in UPR

We next analyzed the chromosomal distribution of the lncRNAs differentially expressed greater than twofold after UPR activation, and found that these transcripts scattered widely on all chromosomes (Supplementary Fig. 2). Several recent studies have suggested that the expression patterns of many lncRNAs strongly correlate with their neighboring/overlapping coding genes^8^. We therefore examined the presence of any coding genes neighboring or overlapping with the most differentially expressed lncRNAs identified in the microarray study. We found that, among the lncRNAs differentially expressed over 5 fold, 22 lncRNAs neighbor or overlap with 22 coding genes, whose expressions also significantly changed in UPR (Table 1). These 22 lncRNAs include one lincRNA (n290468), nine intronic lncRNAs (FR322715, FR342061, FR315668, FR197614, FR351780, FR333518, FR378244, FR201427 and FR262136), nine exonic sense lncRNAs (FR223709, FR167504, n285450, n281650, FR229754, FR086606, FR095406, FR378356 and FR366793), one exonic antisense lncRNA (FR159674) and two overlapping lncRNAs (n295470 and n266048). The expressions of most lncRNAs in this group changed in the same direction as their nearest genes, with comparable fold.

**Table 1:** The information of the nearest genes neighboring/overlapping with the most differentially expressed lncRNA in UPR.

Class	Name	Fold	Gene	Fold
**LincRNA**				
linc	n290468	9.587↓	Adam12	8.534↓
**Intronic lncRNA**				
intronic	FR322715	5.741↓	Pls3	2.763↓
intronic	FR342061	7.093↓	Cacna1c	2.037↓
intronic	FR315668	5.858↓	Pde8b	2.548↓
intronic	FR197614	6.230↓	Errfi1	5.129↓
intronic	FR351780	5.878↓	Cacna1c	2.037↓
intronic	FR333518	6.742↓	Cacna1c	2.037↓
intronic	FR378244	5.092↓	Dlc1	3.857↓
intronic	FR201427	8.457↓	Cacna1c	2.037↓
intronic	FR262136	5.863↑	Cxadr	5.723↑
**Exonic sense lncRNA**				
exonic-sense	FR223709	10.041	Krt20	2.886↓
exonic-sense	FR167504	6.0154↓	Sh3kbp1	2.590↓
exonic-sense	n285450	7.199↓	Mical2	5.734↓
exonic-sense	n281650	5.298↑	Prss16	4.923↑
exonic-sense	FR229754	8.031↑	Sel1l	11.547↑
exonic-sense	FR086606	11.709↓	Has2	15.767↓
exonic-sense	FR095406	9.1600↓	Grb14	4.319↓
exonic-sense	FR378356	5.142↓	Kif26b	4.729↓
exonic-sense	FR366793	5.532↑	Slc7a11	7.919↑
**Exonic antisense lncRNA**				
exonic-antisense	FR159674	5.849↓	Nudt6	2.568↓
**Overlapping lncRNA**				
overlapping	n295470	5.385↓	Igfbp5	4.821↓
overlapping	n266048	6.490↓	Mylk	4.888↓

**Figure 4:**
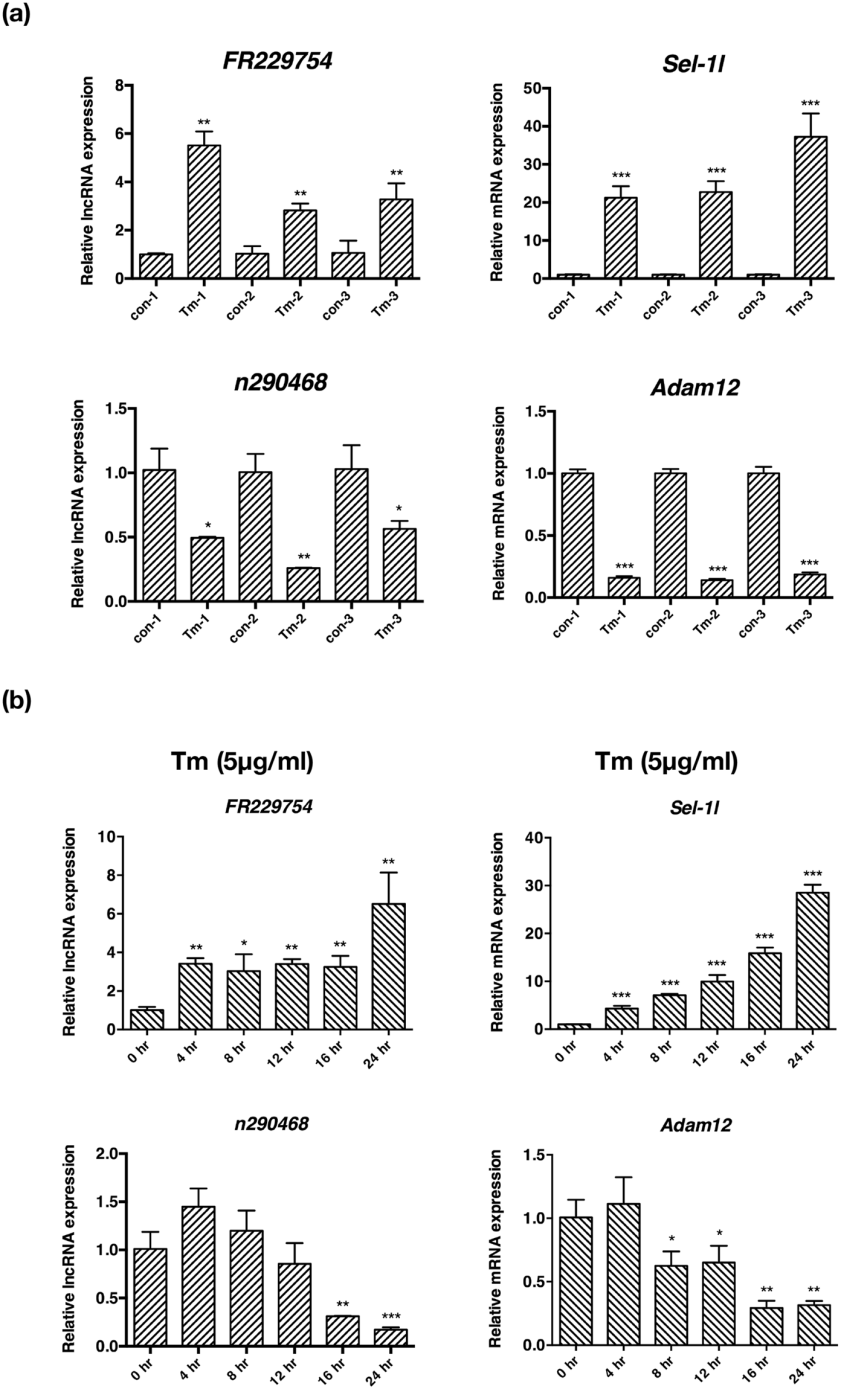
Real-time PCR analysis of lncRNAs and mRNAs identified from genomic location analyses. (**a**) Real-time PCR analysis of FR229754, *Sel1l*, n290468 and *Adam12* in three independent sets of DMSO (control) and tunicamycin (Tm)-treated MEFs at 16 h. (**b**) The expression of FR229754, *Sel1l*, n290468 and *Adam12* over the course of UPR for up to 24 h as analyzed by real-time PCR analysis. All examined RNAs were normalized to *Gapdh*. Data are shown as mean + SD. *P < 0.05, **P < 0.01 and ***P < 0.001. n = 3.

Notably, the nearest gene to an exonic sense lncRNA, FR229754, is *Sel1l*, which encodes the mammalian homologue of yeast Hrd3p. *Sel1l*, which encodes the mammalian homologue of yeast Hrd3p. Hrd3p is a binding partner of Hrd1p, an E3 ubiquitin ligase essential in the ERAD and highly up-regulated in UPR (Fig. 2b). *SEL1L* knockout cells are defective in the degradation of misfolded ER luminal proteins^27^. The expressions of FR229754 and *Sel1l* were up-regulated over 8 and 11 fold in UPR, respectively, as shown by the microarray analysis. The rest lncRNA-neighbouring/overlapping genes include *Adam12*, *Pls3*, *Cacna1c*, *Pde8b*, *Errfi1*, *Dlc1*, *Cxadr*, *Krt20*, *Sh3kbp1*, *Mical2*, *Prss16*, *Has2*, *Grb14*, *Kif26b*, *Slc7a11*, *Nudt6*, *Igfbp5* and *Mylk*, respectively, which encode proteins involved in a wide spectrum of cellular functions from cytoskeleton organization, ion transport, glucose metabolism to extracellular matrix formation. For example, *Adam12*, which encodes a disintegrin and metalloprotease involved in cell adhesion and extracellular matrix formation, overlaps with a lincRNA, n290468. Hyaluronan synthase 2 (*Has2*) is involved in hyaluronan synthesis and extracellular matrix formation, and is next to an exonic sense lncRNA, FR086606. Real-time PCR and immunoblotting analysis confirmed the transcriptional up-regulation of FR229754 and *Sel1l* and down-regulation of n290468 and *Adam12* in three independent sets of tunicamycin-treated MEFs (Fig. 4a), their trend of change over the course of UPR for up to 24 h (Fig. 4b), and the up-and down-regulation of *Sel1l* and *Adam12* at protein level (Supplementary Fig. 3).

### lncRNA functions predicted by lncRNA-mRNA co-expression analyses

**Figure 5:**
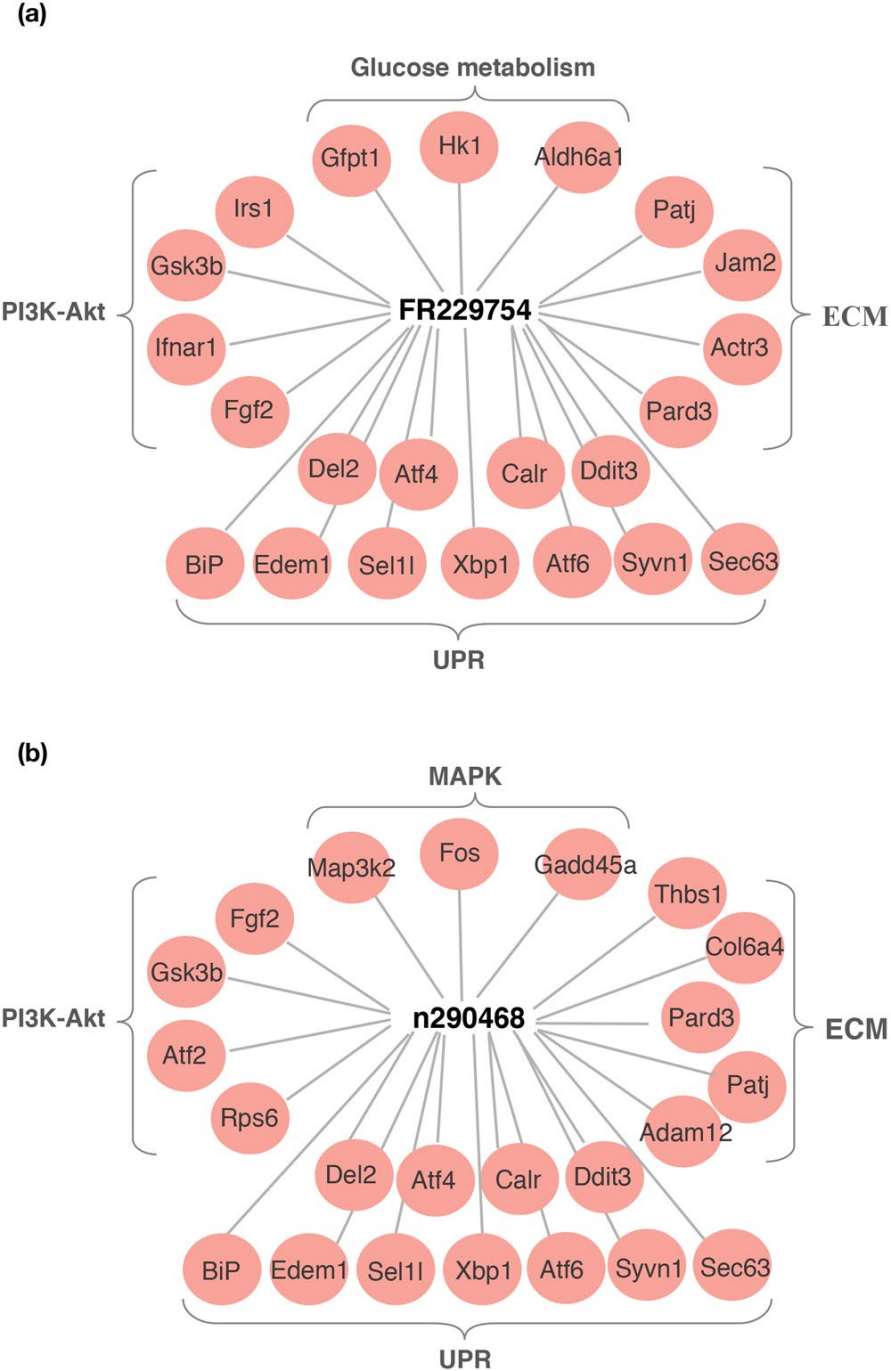
Possible mRNA candidates identified from lncRNA-mRNA co-expression analyses. Representative co-expressing mRNAs identified from lncRNA-mRNA co-expression analyses are depicted in the periphery with FR229754 (**a**) and n290468 (**b**) depicted in the center.

We next identified the mRNAs that were expressed in a fashion statistically correlated with the above-identified lncRNAs, using lncRNA-mRNA co-expression analysis. The identified mRNAs might be expressed under the same promoter, i.e. in the comparable trend as the lncRNA, or represent the possible targets that were regulated by the lncRNA. FR229754 and n290468, which overlaps with *Sel1l* and *Adam12*, respectively, were co-expressed with a large number of UPR genes, in addition to genes and from other cellular pathways, e.g. extracellular matrix (ECM), PI3 kinase (PI3K)-Akt, MAP kinase (MAPK) and glucose metabolism (Fig. 5a,b). FR229754 correlated with the expression of many UPR genes, including several ER chaperones and ERAD components, e.g. *Sel1l*, *BiP*, *Edem*, *Ero1*, *DnaJ* and *Derl3* (Fig. 5a). n290468 was also co-expressed with many UPR genes and several ECM genes including *Adam12* (Fig. 5b).

### The functional impact and expression of FR229754

**Figure 6:**
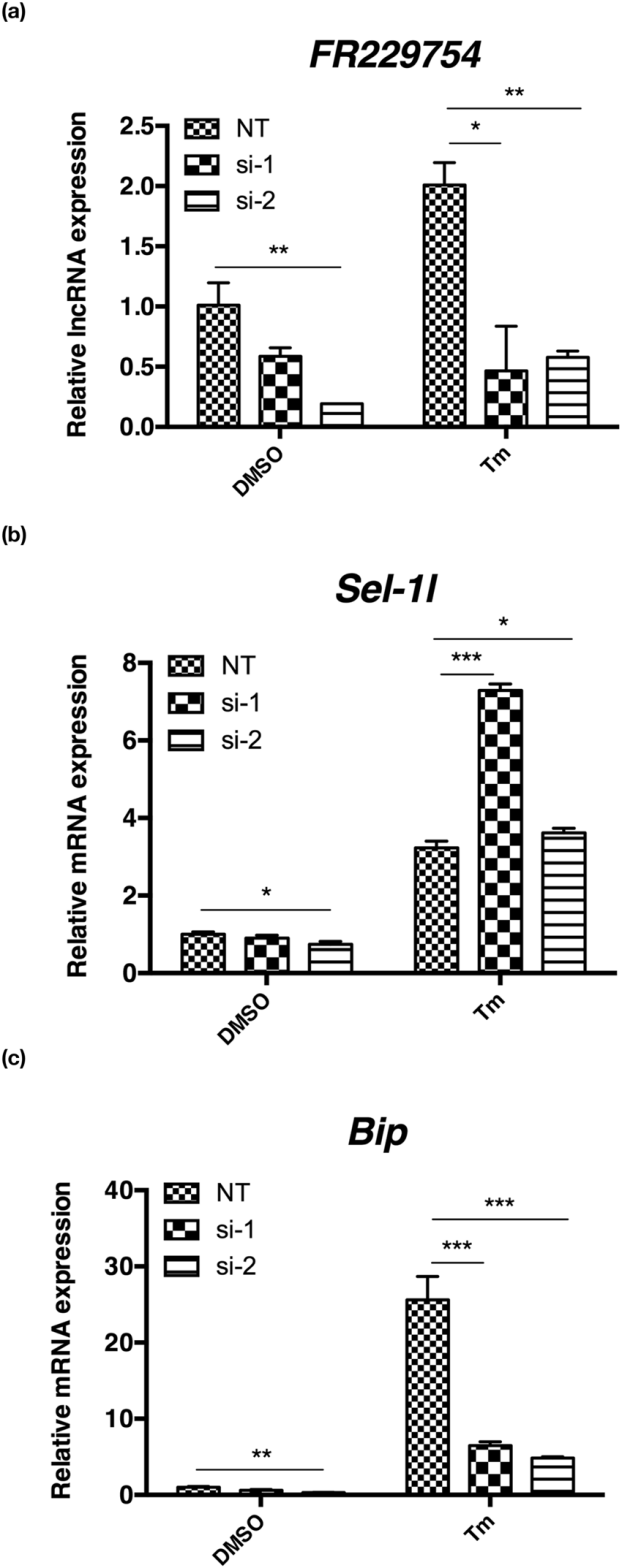
Silencing of FR229754 markedly reduced *BiP* levels. (**a–c**) Real-time PCR analysis of FR229754, *Sel1l* and *BiP* in the DMSO and tunicamycin-treated MEFs, transfected with negative control non-targeting (NT) and two small interfering (si-1 and si-2) RNAs designed against FR229754. Note that si-2 is more potent in silencing FR229754 in the MEFs. All examined RNAs were normalized to *Gapdh*. Data are shown as mean + SD. *P < 0.05, **P < 0.01 and ***P < 0.001. n = 3.

Because the expression of FR229754 correlated with *Sel1l* and many UPR genes, and it was strongly up-regulated in UPR, we next examined the impact of FR229754 silencing on the expression of *Sel1l* and *BiP*. Two small interfering RNAs designed against FR229754 reduced the expression of FR229754 in the unstressed and tunicamycin-treated MEFs, with si-2 being more potent (Fig. 6a). Interestingly, the level of *Sel1l* was not much affected by FR229754 in the MEFs transfected with si-2, while the level of *BiP* was greatly reduced and especially in the tunicamycin-treated MEFs transfected with si-2, suggesting that the level of FR229754 affects the up-regulation of *BiP* in UPR (Fig. 6b,c).

**Figure 7:**
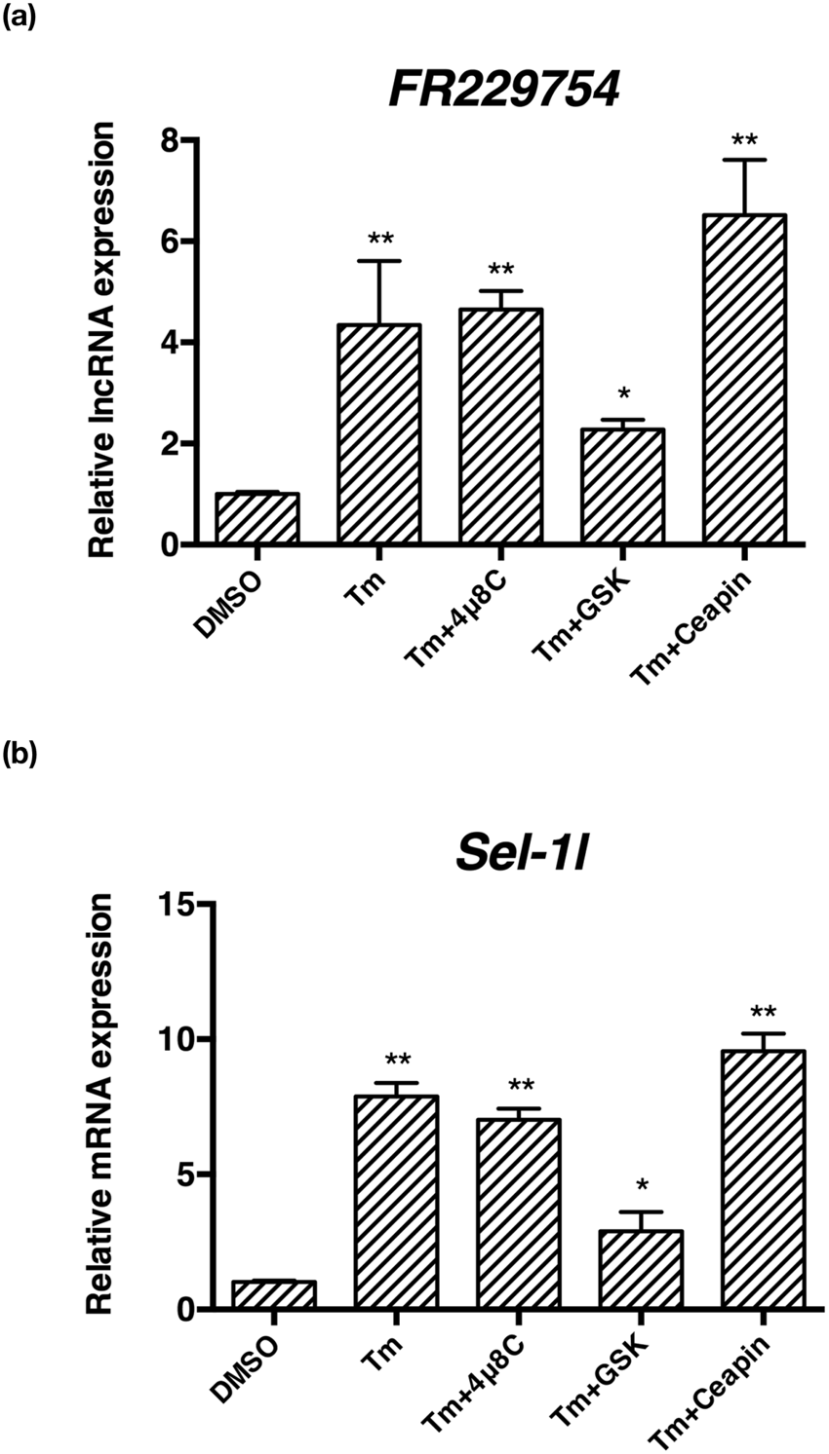
The involvement of the three UPR branches in FR229754 expression. MEFs were treated with tunicamycin (5 μg/ml) for 16 h and in the presence of 10 μM 4μ8C, 10 μM GSK2606414 and 10 μM CEAPIN-A7 to assess the contribution of Ire1α, Perk and Atf6α. (**a,b**) Real-time PCR analysis of FR229754 and *Sel1l*. All examined RNAs were normalized to *Gapdh*. Data are shown as mean + SD. *P < 0.05, **P < 0.01 and ***P < 0.001. n = 3.

We further analyzed which UPR branch was more intimately involved in the expression of FR229754 and *Sel1l*. In order to assess the contribution of each UPR branch (Ire1α, Perk and Atf6) to lncRNA expression, we used the specific inhibitors targeting Ire1α, Perk and Atf6α, respectively. 4μ8C, GSK2606414 and Ceapin-A7, inhibit the *XBP1* splicing, PERK phosphorylation and the ER-Golgi trafficking of ATF6, respectively^28–30^. Several studies have suggested that the transcriptional regulation of UPR target genes relies on the activation of individual UPR branch, i.e. Ire1α-, Perk- and Atf6α-dependent^26,31,32^, although overlap in some target genes has also been reported^25^. Real-time PCR analysis (Supplementary Fig. 4) confirmed that treatment with 4μ8C, GSK2606414 and Ceapin-A7 induced similar changes to many UPR gene [including ER chaperones (*BIP*), PERK downstream effector (*Chop*, *ATF4*) and ERAD effector (*EDEM* and *p58*)] expression, in a comparable fashion as the IRE1α-, PERK- and ATF6α-deficient MEFs^25^. In our experiment, incubation with GSK2606414, not 4μ8C and Ceapin-A7, largely reversed the increase of FR229754 and *Sel1l* induced by tunicamycin treatment, suggesting that the activation of Perk branch, not Ire1α and Atf6α, mediates the transcriptional up-regulation of FR229754 and *Sel1l* (Fig. 7a,b).

## Discussion

In this study, we examined the transcriptomic profiles of lncRNAs and mRNAs of MEFs treated with ER stress-inducing compound. Our data of the mRNA profiles in MEFs treated with tunicamycin are consistent with previous profiling studies in MEFs and other tissues^33–37^. Interestingly, we found that genes from pathways seemingly unrelated to the ER were also significantly altered after UPR activation. These include genes responsible for cell adhesion and extracellular matrix remodeling, e.g. *Adam12*, *Has2* and collagen isoforms (*CO1A1*, *CO1A2* and *CO6A1*). Similar findings were reported by a proteomics study^36^. Our lncRNA-mRNA co-expression analysis also showed that FR229754 and n290468 were co-expressed with genes from other cellular pathways such as ECM, PI3K-Akt, MAPK and glucose metabolism. These results strongly suggest that the activation of UPR induces the adaptive changes in multiple cellular processes, in addition to the canonical ER proteostasis-regulating pathway. How the canonical and noncanonical pathways coordinate in the activation and maintenance of UPR awaits future characterization. Some lncRNAs identified in this study may link these pathways together.

We used two approaches to probe the potential functions of the differentially expressed lncRNAs in UPR. First, by identifying the genomic location of the most differentially expressed lncRNAs, we predicted that the genes, which neighbor or overlap with the differentially expressed lncRNAs, may be their cis targets. On the other hand, we used the lncRNA-mRNA network to identify the mRNAs, whose expressions correlated with two differentially expressed lncRNAs, FR229754 and n290468. The identification of *Sel1l* and *Adam12* as their co-expressing mRNAs corroborated our genomic location approach. A host of lncRNAs are known to regulate the expressions of their target genes at epigenetic (as recruiters, tethers and scaffolds) and transcriptional (as decoys, coregulators, and RNA polymerase II inhibitors) levels^8^. Future studies are required to characterize how the lncRNAs identified by these two approaches mechanistically affect the expression of their potential target genes, including UPR-related and unrelated genes.

One interesting lncRNA candidate identified from this study is FR229754, which overlaps with *Sel1l* in terms of genomic sequence and correlated with *Sel1l* and other UPR genes such as *BiP* at expression level. Studies in yeast and higher organisms have identified three different pathways for the degradation of ERAD substrates, depending on the location of the misfolded domains, namely ERAD-L, ERAD-M and ERAD-C (with misfolding in the ER lumen, inside the ER membrane and on the cytosolic side of a transmembrane protein, respectively)^38^. The ERAD-L pathway is further categorized into the ERAD-Ls (for degradation of soluble luminal proteins) and ERAD-Lm (degradation of transmembrane proteins) pathways^39^. In yeast and higher organisms, Hrd1p is shown to is shown to recognize and degrade ERAD-L and ERAD-M substrates, whereas the other evolutionarily conserved ER ubiquitin ligase Doa10/TEB4 mediates ERAD-C substrate degradation^38–42^.

Mammalian and chicken Sel1l have been shown as an essential component required for the degradation of ERAD-Ls, but not ERAD-Lm substrates^27,39–41^. *Sel1l* knockout mice suffer embryonic lethality^43^. Silencing of FR229754 greatly reduced the level of *BiP*, indicating that FR229754 level affects the chaperone adaptive response in UPR. We further used specific inhibitors and demonstrated that the transcriptional up-regulation of FR229754 and *Sel1l* is dependent on the activation of Perk branch. Because the inhibitors used to block the three UPR pathways likely have off-target effects, future studies using the MEFs isolated from IRE1α-, PERK- and ATF6α-deficient mice would be required to validate and compare with the findings of our study. In summary, our study identified a large number of differentially expressed lncRNAs after the UPR activation. The lncRNAs and the possible mRNA targets predicted by the bioinformatic analysis in this study lay a foundation for future functional characterization of non-coding RNAs in UPR.

## Methods

### Animals

Pathogen-free male and female C57BL/6 J mice were purchased from Vital River Laboratory Animal Technology Co. Ltd. (Beijing, China). 10-week-old mice were mated in a temperature-controlled room with a 12-h light/dark cycle. Pregnancy was assessed by visual inspection of a distended abdomen. The animal protocol followed the “Principles of laboratory animal care” (NIH publication No. 86-23), and was approved by the Ethics Committee of Peking Union Medical College.

### Cell culture and chemicals

Mouse embryonic fibroblasts (MEFs) were isolated from pregnant mice at E13.5 and cultured in DMEM containing 10% fetal bovine serum as previously described^44^. Tunicamycin, thapsigargin, 4μ8C and GSK2606414 were purchased from Calbiochem. Ceapin-A7 was a generous gift from Drs. Ciara Gallagher and Peter Walter (University of California San Francisco). All other chemicals were purchased from Sigma-Aldrich, unless stated otherwise. To induce ER stress, MEFs were treated with 5 μg/ml tunicamycin (Tm) for up to 24 hours. To inactivate each UPR branch (Ire1α, Perk and Atf6α), the MEF cells were treated with 10 μM 4μ8C, 10 μM GSK2606414 and 10 μM Ceapin-A7.

### RNA extraction and reverse transcription-polymerase chain reaction

Total RNAs were extracted from cells using Trizol (Invitrogen, USA), following the manufacturer’s instruction. The concentration and purity of the RNA samples were determined using a NanoDrop 2000C (Thermo Scientific, USA). 1.5 μg of RNA was reverse transcribed to cDNA using oligo(dT) and random primers with the TransScript First-Strand cDNA Synthesis SuperMix kit (TransGen Biotech, Beijing). The *Xbp1* splicing was detected by standard reverse transcription-polymerase chain reaction using 2 × Taq PCR StarMix (GenStar, Beijing). The specific primers for murine *Xbp1* and Glyceraldehyde-3-phosphate dehydrogenase (*Gapdh*) are as follows: *Xbp1*: Forward, GAACCAGGAGTTAAGAACACG; Reverse, AGGCAACAGTGTCAGAGTCC; *Gapdh*: Forward, GGCCTCCAAGGAGTAAGA; Reverse, GTGCAGCGAACTTTATTGA.

### Microarray analysis

The OE Mouse lncRNA Microarray V2.0 (OEBiotech, Shanghai) was used for the global profiling of mouse lncRNAs and protein-coding transcripts. Total RNAs were quantified and the RNA integrity was assessed using Agilent Bioanalyzer 2100 (Agilent Technologies, USA). The labeling, microarray hybridization and wash were performed by the microarray facility at OEBiotech, Shanghai. Briefly, cDNAs were transcribed from total RNAs, synthesized to cRNAs and labeled with cyanine-3-CTP. The labeled cRNAs were then hybridized onto the microarray. After wash, the fluorescent signals were scanned using the Agilent Scanner G2505C (Agilent Technologies, USA).

Feature Extraction software V10.7.1.1 (Agilent Technologies, USA) was used to analyze the array images to obtain the raw data, which was further analyzed by Genespring software (Agilent Technologies, USA). The differentially expressed protein-coding genes and lncRNAs were identified, and fold change as well as P value from the statistics t-test were calculated. We set a limit for the up- or down-regulated lncRNAs as fold change ≥ 2.0 and P value ≤ 0.05.

### Real-time quantitative PCR (Real-time PCR)

Primers for mRNA or lncRNA used in real-time PCR were designed using Primer 6 software (PREMIER Biosoft, USA), and then verified using the Basic Local Alignment Search Tool (BLAST) from National Centre for Biotechnology Information. Sequence information is as follows: *Ire1α*: Forward, 5′-TTCTGAGGTTCTTAGCCA-3′; Reverse, 5′-CATGCATTCACAAACATGA-3′; *Atf4*: Forward, 5′-CCTGATAGAAGAGGTCCG-3′; Reverse, 5′-GGTACTTTCACTACAAAATAAT-3′; *Perk*: Forward, 5′-ATTTATGTCGGTAGTGTCA-3′; Reverse, 5′-CTTGAAAGAAGTCATAATAGTT-3′; *BiP*: Forward, 5′-CAGAGTGGAGTTGAAAAT-3′; Reverse, 5′-AAAATTAGACCAGTGTAAA-3′; *Chop*: Forward, 5′-CCTGCCTTTCACCTTGGA-3′; Reverse, 5′-GCTTTGGGATGTGCGTGT-3′; *Atf6α*: Forward, 5′-TGAGCAGCTGAAGAAGGAGA-3′; Reverse, 5′-TTCTCTGACACCACCTCGTC-3′; *Sel1l*: Forward, 5′-TTCGTCTGGCTGCTTGGT-3′; Reverse, 5′-TGCGTATCTTCTTGCGTGTT-3′; *Adam12*: Forward, 5′-TGGGACCAGAGAGGAGCTTAC-3′; Reverse, 5′-GTTGCACAGTCAGCACGTCT-3′; *Has2*: Forward, 5′-GAGCACCAAGGTTCTGCTTC-3′; Reverse, 5′-CTCTCCATACGGCGAGAGTC-3′; *Krt20*: Forward, 5′-GTGGCTCGCTGTATAGGAAGG-3′; Reverse, 5′-CAGGTCCGATCCGTTGGAG-3′; *Hrd1*: Forward, 5′-AGCTACTTCAGTGAACCCCACT-3′; Reverse, 5′-CTCCTCTACAATGCCCACTGAC-3′; *Edem*: Forward, 5′-TCTACATGCGCCAGATCGAC-3′; Reverse, 5′-TCGACAGCATCACAGATGGG-3′; *Ire1β*: Forward, 5′- TGAGGAACAAGAAGCACCACT-3′; Reverse, 5′- AGAGCTGGTGGGTAGTAGGG-3′; *Atf6β*: Forward, 5′- GAGGAGCAGGCACAGTTGTT-3′; Reverse, 5′- AAGATGGGTAGAGGGTCCCA-3′; *p58ipk*: Forward, 5′-GCTGAACTCCGTTCTGTCCA-3′; Reverse, 5′-TCGACAGCATCACAGATGGG-3′; *Calnexin*: Forward, 5′-GCTAGGGAGAATGAATTGCCG-3′; Reverse, 5′- TTGGGCTTCCATCCAATCGC-3′; *Calreticulin*: Forward, 5′-TACAAGGGCGAGTGGAAACC-3′; Reverse, 5′-GCATCGGGGGAGTATTCAGG-3′; *Gapdh*: Forward, 5′-CCCAACACTGAGCATCTCC-3′; Reverse, 5′-GGGTGCAGCGAACTTTATT-3′*;* FR223708: Forward, 5′-ATTAAAGCAGTTACACCCAGCA-3′; Reverse, 5′-ACCCAACGCTACCATCCAC-3′; FR229754: Forward, 5′-GGTCGCCCTGCCCACA-3′; Reverse, 5′-AAACCCCAGCGTGTCCC-3′; FR091011: Forward, 5′-AGGACTAGAGTAAGCAGGAGA-3′; Reverse, 5′-TTCACGGCTGTGGGTTGA-3′; FR346657: Forward, 5′-GTGCGGTGGTTACAGAAG-3′; Reverse, 5′-CAGTCCCAGGTGGCATCC-3′; n290468: Forward, 5′-AGCCAAACACTCAACTGGAC-3′; Reverse, 5′-GGCCTCCTTTCCAACATGCTT-3′; n416682: Forward, 5′-AAGGCAAACCAAACAGAC-3′; Reverse, 5′-TACAGCACGGATGAAGAG-3′; n290844: Forward, 5′-GCGTAGAGGGCAGGTATTGT-3′; Reverse, 5′- CAGGAATCAGACCCACGGAA-3′; n278914: Forward, 5′-GTGGCCCAGATCTTTCCATCT-3′; Reverse, 5′-AAGGCCAGACTGGGGAGAAG-3′. The real-time PCR reactions were performed in 96-well optical plates using StepOnePlus^TM^ Real-time PCR system (Applied Biosystems) with SYBR Green PCR Master Mix (TransGen Biotech, Beijing). All experiments were performed in triplicate, and all samples were normalized to the expression of *Gapdh*.

### Genomic sequence analysis

The genomic sequence of murine lncRNAs and mRNAs were obtained using the UCSC genome browser (http://genome.ucsc.edu/) and the following databases: Ensembl, RefSeq, Ultra-conserved region encoding LncRNA (UCR), lncRNAdb, ncRNA and NONCODE. The chromosome locations of lncRNAs were annotated by the version of *Dec. 2011 (GRCm38/mm10)*.

### Construction of the lncRNA-mRNA co-expression network

The lncRNA-mRNA co-expression network was constructed using the normalized signal intensity of each differentially expressed lncRNA identified from the microarray study against the normalized signal intensity of every mRNA. The Pearson correlation was calculated for each lncRNA-mRNA pair, and then the pairs with correlation coefficient > 0.99 and *P*-value < 0.05 were deemed significant.

### Gene ontological analysis

Gene ontology (GO) (http://www.geneontology.org) was used to assign the co-expressed mRNAs to GO terms and thereby predict the potential molecular functions and biological processes that one lncRNA may be involved. Briefly, we annotated the GO terms of the co-expressed mRNAs for each lncRNA, and then conducted a functional enrichment for the co-expressed mRNAs by summating the GO terms. The enriched functional terms were used to access the involvement of one given lncRNA in biological processes, cellular components and molecular functions. KEGG pathway analyses were performed to predict the biological pathways (http://www.genome.ad.jp/kegg/).

### Immunoblotting

Total cellular proteins were prepared using a lysis buffer [20 mM HEPES pH7.5, 150 mM NaCl, 1% Triton-X100, 10% glycerol, 1 mM EDTA 10 mM tetrasodium pyrophosphate, 100 mM NaF, 17.5 mM β-glycerophosphate, 1 mM phenylmethysulfonyl fluoride and protease inhibitor cocktail (Roche)]. The protein lysates were analyzed by SDS-polyacrylamide gel electrophoresis (SDS-PAGE), electrotransferred to polyvinylidene difluoride (PVDF) membranes, and then subjected to immunoblotting using a standard protocol. The primary antibodies used in this study include: PARP (Cell Signaling Technology, 9542), Adam12 (Proteintech, 14139-1-AP), Sel-1l (Santa Cruz, sc-377350), β-actin (Molecular Biological Laboratories, PM053), GAPDH (Proteintech, 60004-1-Ig). After incubation with peroxidase-conjugated secondary antibody (anti-Rabbit IgG, 074-1506, KPL; anti-Mouse IgG, 330, Molecular Biological Laboratories), the proteins of interest were visualized with Clarify Western ECL Substrate (Bio-Rad).

### RNA interference

RNA interference was performed using RNAiMAX (Invitrogen). Briefly, MEFs were seeded in 6-well plates and cultured overnight, and transfected with siRNA oligonucleotides (50 nmol per well) with RNAiMAX according to the manufacturer’s instruction. 48 h after transfection, cells were harvested for further analysis. The siRNA oligonucleotides were designed using an on-site algorithms and synthesized by GenePhama (Shanghai, China) as follows: *FR229754*: siRNA-1: 5′-GCAGCAAACUUUAGGUGAC-3′; siRNA-2: 5′-UCGACUAGCUGACUUACAU-3′. Non-targeting (NT) siRNA: 5′-UUCUCCGAACGUGUCACGU-3′ was also purchased from GenePhama (Shanghai, China).

### Statistical analysis

Expression ratios were subjected to a log_2_ transformation to produce fold-change data. Differential expression levels of lncRNAs and mRNAs were compared, using an independent sample t-test between two groups. *P* < 0.05 was considered significant.

## Electronic supplementary material

Supplementary Figures 1-4
