# Supplementary material for: The transcriptional profiles and functional implications of long non-coding RNAs in the unfolded protein response
Source: Sci Rep. 2018 Mar 21;8:4981. doi: 10.1038/s41598-018-23289-3 (PMC5862980; doi:10.1038/s41598-018-23289-3)
Supplement: Supplementary file 1 — Supplementary Figures 1-4 [file 41598_2018_23289_MOESM1_ESM.pdf]

The transcriptional profiles and functional implications of long non-coding RNAs in the unfolded protein response

Hongyang Quan[1,#], Qianqian Fan[1,#], Chuang Li[1,#], Yan-Ying Wang[1] and Lin Wang[1,*]

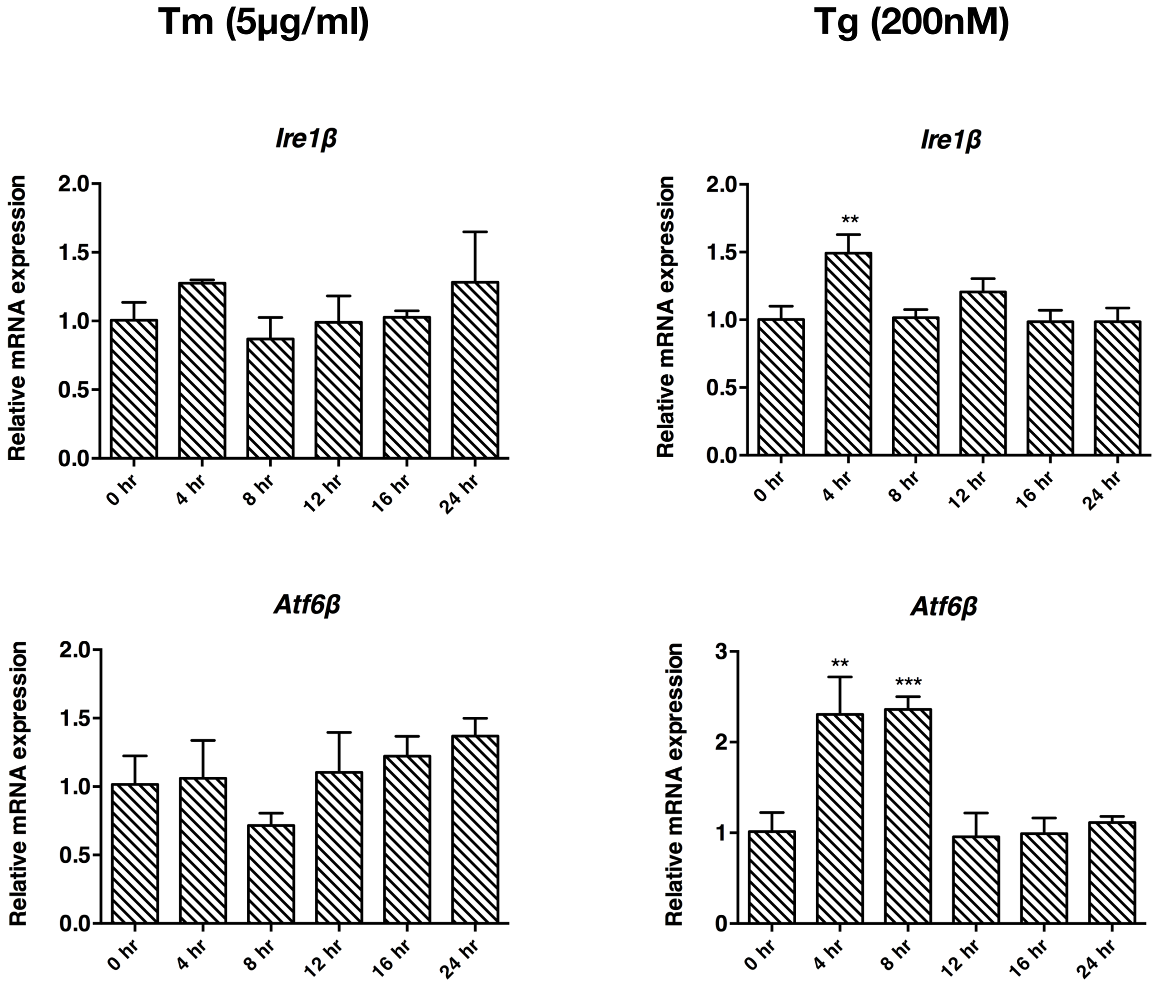

Supplementary Figure 1. Real-time PCR analysis of *IRE1β* and ATF6*β* levels in MEFs.
MEFs were treated with 5 µg/ml tunicamycin (Tm) or 200 nM thapsigargin (Tg) with DMSO as a vehicle control as indicated. All examined mRNAs were normalized to *Gapdh*. Data are shown as mean + SD. *$P < 0.05$, **$P < 0.01$ and ***$P < 0.001$. $n = 3$.

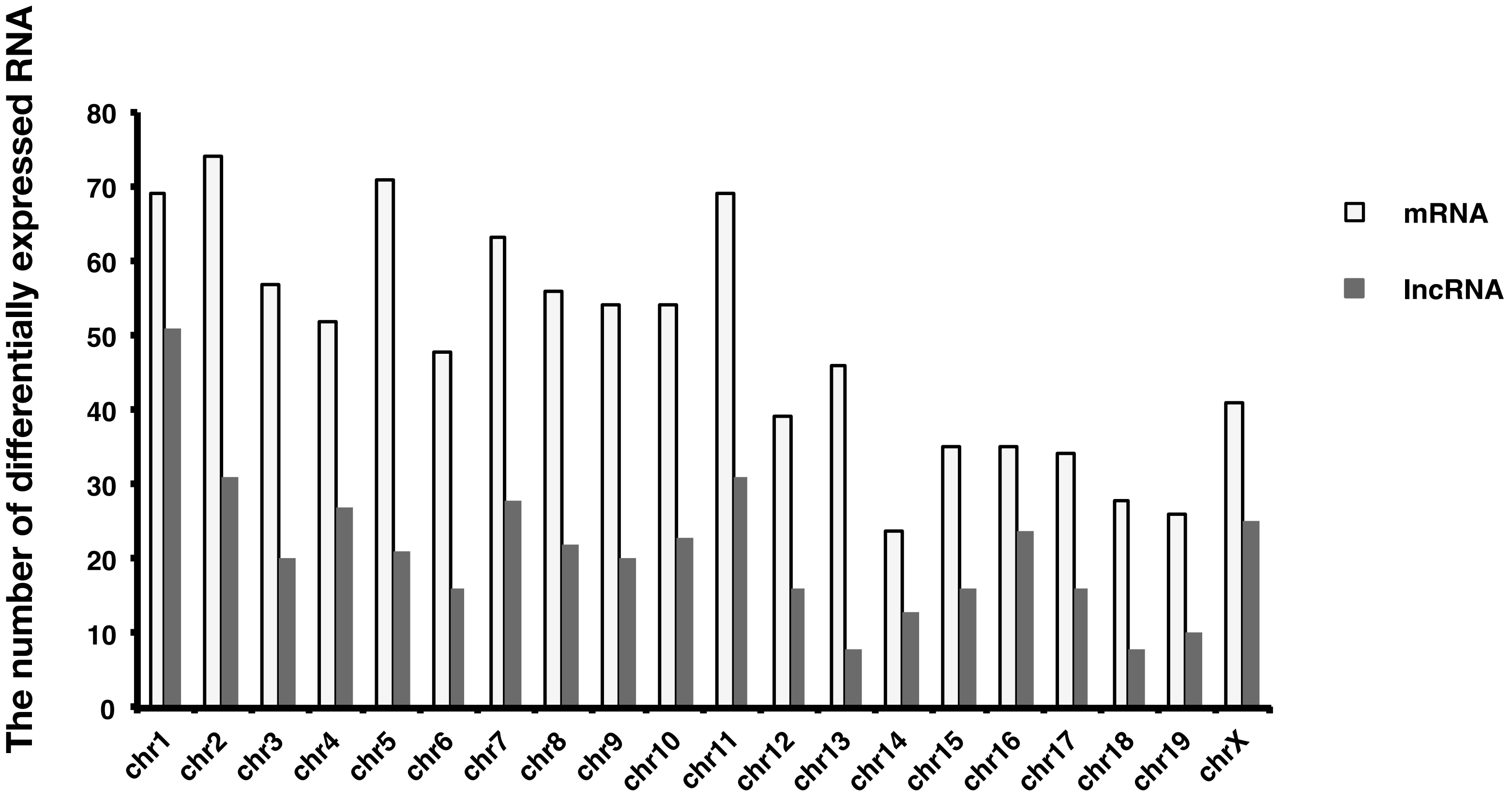

Supplementary Figure 2. The number of differentially expressed mRNA and lncRNA greater than twofold on each chromosome after UPR activation.

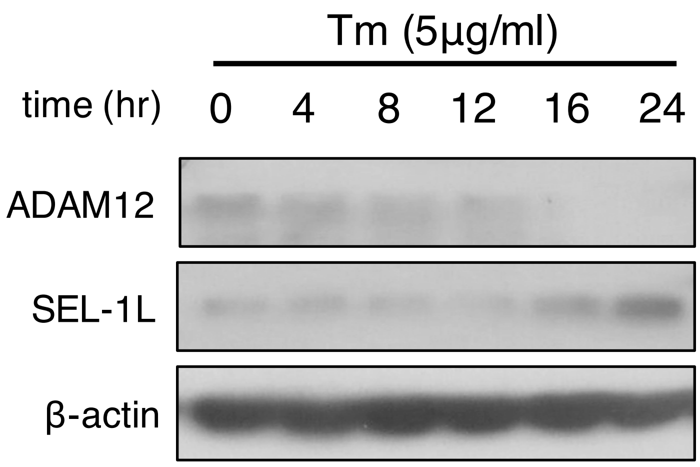

Supplementary Figure 3. Immunoblotting analysis of SEL-1L and ADAM12 in MEFs treated with 5 µg/ml tunicamycin (Tm) with DMSO as a vehicle control for the indicated time. β-actin serves as a loading control.

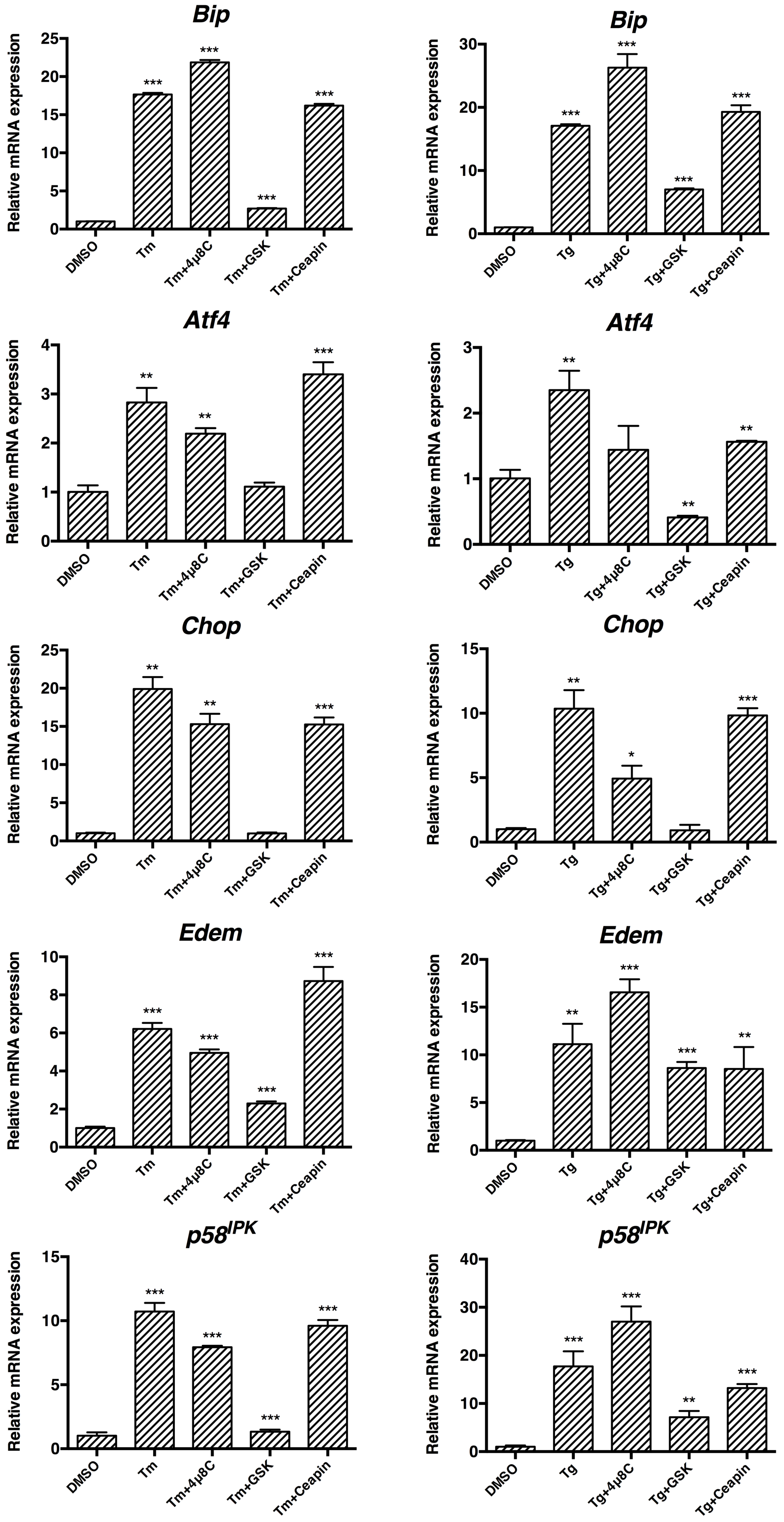

Supplementary Figure 4. Real-time PCR analysis of UPR genes in MEFs.
MEFs were treated with tunicamycin (5 µg/ml) or 200 nM thapsigargin (Tg) and in the presence of 10 µM 4µ8C, 10 µM GSK2606414 and 10 µM CEAPIN-A7 to assess the contribution of Ire1α, Perk and Atf6α. All examined mRNAs were normalized to *Gapdh*. Data are shown as mean + SD. $^{*}P < 0.05$, $^{**}P < 0.01$ and $^{***}P < 0.001$. n = 3.

uncropped images of figure 1c

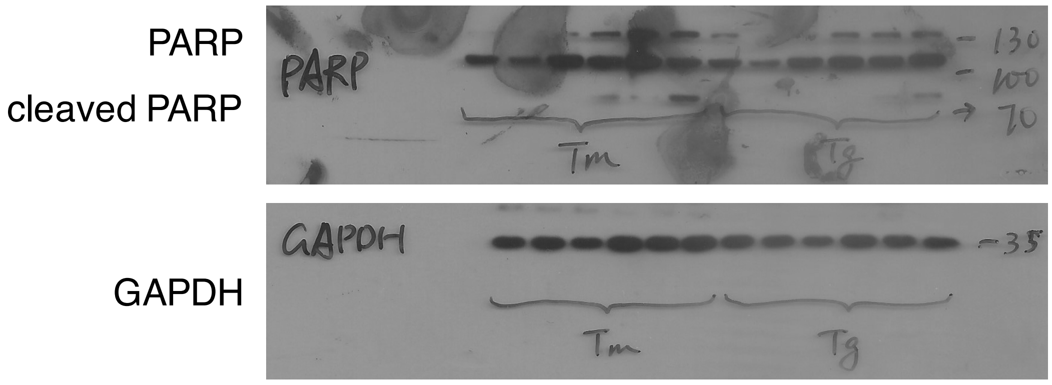

uncropped images of supplementary figure 3

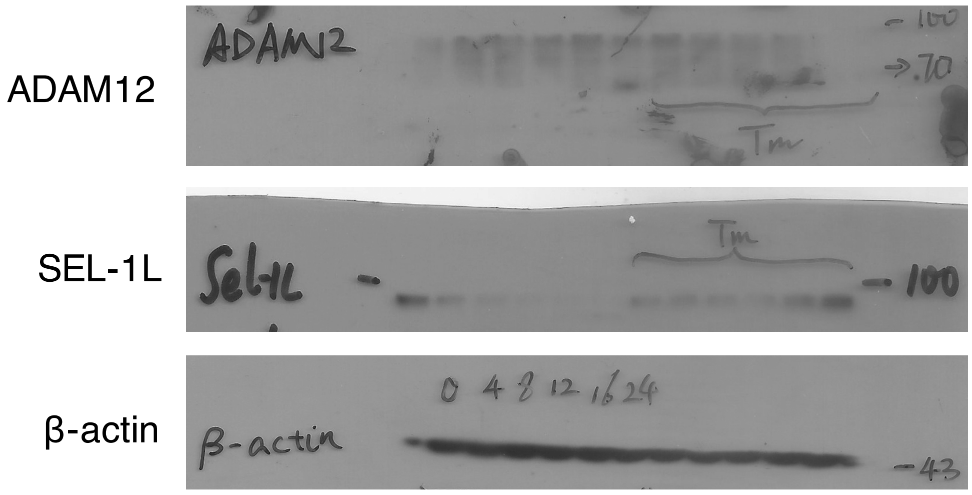

Supplementary Figure 5. The original scanning images for immunoblotting experiments. The original, uncropped scanning images for the immunoblotting experiments as indicated.
